# Carrabiitol^®^, a Novel Oligosaccharide Polyol Composition, Mitigates the Impact of Flooding, Drought, Salinity, and High Temperature in Tomato

**DOI:** 10.3390/biology13050356

**Published:** 2024-05-19

**Authors:** Femida Yunus Patel, Kaushal Kishore Upreti, Ramanna Hunashikatti Laxman, Neil Jaykumar Shah

**Affiliations:** 1 Agri Biochem Research Lab, M/s. Pushpa J. Shah, GIDC Panoli, Ankleshwar 394116, India; 2 Division of Biosciences, Indian Institute of Horticulture Research, Indian Council of Agriculture Research, Bengaluru 560089, India

**Keywords:** *Solanum lycopersicum*, abiotic, osmolytes, sustainable agriculture

## Abstract

**Simple Summary:**

The present research work aimed to study the influence of Carrabiitol^®^, an oligosaccharide polyol composition, in alleviating the adverse impact of various abiotic stresses in tomato (*Solanum lycopersicum* L.) plants. Experiments included raising tomato plants treated with different dosages of Carrabiitol^®^ and studying their growth and physiological and biochemical parameters on exposure to abiotic stresses, namely salinity, flooding, drought, and high temperatures. It was observed that plants that were raised from pre-treated seeds or those given booster dosages at the 2–3 leaf stage or at the flowering stage were more resistant to the adverse effects of the abiotic stresses. It can be concluded that the Carraiitol^®^ formulation influences the growth, physiological, and biochemical parameters of tomato plants grown under stress conditions. The formulation can serve as an effective, sustainable, and eco-friendly biostimulant for plant growth and productivity and is very relevant in the present scenario of climate change.

**Abstract:**

Abiotic stress results in various physiological and biochemical changes in plants. Osmolytes play a pivotal role in improving the tolerance to abiotic stress in plants. This study evaluated the effectiveness of a commercial formulation, Carrabiitol^®^, an oligosaccharide polyol composition, in alleviating adverse impacts of abiotic stress in tomato (*Solanum lycopersicum* L. var. *Arka Rakshak*) plants. Plants were raised from seed and treated with 1 mL/L, 2 mL/L, and 3 mL/L of Carrabiitol^®^. The foliage of developing plants was treated at the 2–3 leaf stage (T2, T3, and T4) and at pre-flowering stage (T5, T6, and T7). Growth conditions were compared with those of plants developed from untreated seed (T1). Developing tomato plants were then exposed to flooding, salinity (50 mM NaCl), high temperature (41.1 °C), or drought at the flowering stage. Plants were evaluated for their dry weight, leaf water potential, stomatal conductance, transpiration rate, antioxidant potential, chlorophyll, carotenoid, glucose, sucrose, malondialdehyde, and proline contents. Pre-treated seed, which received a booster treatment at the 2–3 leaf stage (T4 = seed treatment and booster at the 2–3 leaf stage with 3 mL/L Carrabiitol^®^) and pre-flowering stages (T5, T6, and T7 = seed treatment and booster doses at the pre-flowering stage with 1, 2, and 3 mL/L Carrabiitol^®^, respectively), was effective in mitigating negative impacts on various growth parameters of stressed tomato plants (*p* < 0.05). Carrabiitol^®^ may be an effective, sustainable, and bio-rational organic osmolyte formulation for reducing the effects of abiotic stress on plant growth and productivity.

## 1. Introduction

Globally, the demand for agriculture to ensure food and nutritional security has increased day by day [1]. Despite advances in the agriculture sector, the industry faces stress due to global warming and climate change [2]. An increase in arid and semi-arid environments, changes in soil fertility, extreme heat and cold, drought, floods, and salinity are all examples of climate change that have adverse impacts on the development and productivity of plants [3]. 

Plant cells experience osmotic and oxidative stress as a result of drought. The physiological effects of drought include stomata closing, a rise in cellular CO_2_ that enhances photorespiration, and a decrease in photosynthesis. Salinity is caused by large amounts of cations such as Na^+^, Ca^2+^, and Mg^2+^ and lower amounts of K^+^, Fe^2+^, and anions such as Cl^−^, SO_4_^2−^ HCO_3_^−^, etc. [4]. These osmotic imbalances lead to salt stress that reduces water uptake in plants and can cause ionic toxicity in cells, lower the amount of chlorophyll, and produce reactive oxygen species (ROS) [5,6]. High temperatures lead to water loss by transpiration and evaporation [7], leading to drought-like conditions. High soil temperatures lead to a decrease in seed germination and affect 2–3 leaf stage growth and development of plants [8]. 

Increased production of non-enzymatic antioxidants like polyphenols and carotenoids and enzymatic antioxidants like superoxide dismutase, catalase, and peroxidases is yet another response seen towards abiotic stress in plants [9]. In intracellular signaling pathways, ROS serve as secondary messengers, but when they are overproduced under stressed conditions, they cause oxidative damage to cell organelles and cells [10]. As a result, when plants are under stressed conditions, their cellular systems produce antioxidants to combat ROS.

In order to counteract osmotic imbalances brought on by abiotic stress, plant cells produce low-molecular-weight organic molecules known as osmolytes. Osmolytes manage the solute concentration inside a cell by balancing the osmotic potential and preventing the loss of turgor pressure. This facilitates the opening of the stomata and restores physiological functions like transpiration and photosynthesis that are otherwise impeded under abiotic conditions. By removing ROS from the environment and preventing their synthesis, osmolytes also shield plant cells from oxidative stress [8]. 

Numerous examples of such organic osmolytes include sugars, sugar alcohols, amino acids, and polyamines, among others [11,12]. These groups of compounds are recognized to aid plants in overcoming abiotic stress, promote plant growth [12], and, thereby, hold enormous promise. 

Moreover, organic osmolytes are crucial in today’s setting of bio-rational and sustainable farming methods, and their significance is enhanced when sourced or produced from natural resources [13]. Progressively, more research attention is being focused on sustainable sources of organic osmolytes such as polyols, polysaccharides, and oligosaccharides such as fucoidan, alginate, and carrageenan [14,15]. 

Carrabiitol^®^, a novel oligosaccharide polyol composition, was previously evaluated at seed germination and seedling stages on Fenugreek and sorghum under abiotic stresses [16]. The experiment results showed that the application of Carrabiitol^®^ was effective in improving seed germination and seedling growth under drought, salinity, and excess water stress. In view of the above, the aim of the present study was to evaluate the effects of Carrabiitol^®^ on growth, physiological, and biochemical parameters in tomato plants grown under various abiotic stress conditions.

## 2. Materials and Methods

### 2.1. Experimental Design

Carrabiitol^®^, a patented formulation (M/s Pushpa J. Shah, Panoli, India) [17] that acts as an organic osmolyte, is an oligosaccharide polyol. The raw material was treated with organic acid, followed by a metal ion complex, and thereafter neutralized to attain a working formulation. 

‘Arka Rakshak’, a high-yielding variety of tomatoes (*Solanum lycopersicum L.),* was used. Field trials were conducted at the Indian Council of Agriculture Research-Indian Institute of Horticultural Research campus in Hessaraghatta Lake Post, Bangalore, India. Experiments were conducted on plants raised from seed pre-treated with doses of Carrabiitol^®^. The experiments were conducted in two sets to evaluate (A) flooding and salinity and (B) heat and drought. The first set of experiments (for studying flooding and salinity) was carried out from March 2021 to June 2021, and the second set of experiments was carried out beginning July 2021, and data were collected by the end of September of the same year. 

Treatment consisted of seed without Carrabiitol^®^ application (T1), seed treated with 1 mL/L Carrabiitol^®^ and foliar application at 2–3 leaf stage with 1 mL/L Carrabiitol^®^ + stress at flowering stage (T2), seed treated with 2 mL/L Carrabiitol^®^ and foliar application at 2–3 leaf stage with 2 mL/L Carrabiitol^®^ + stress at flowering stage (T3), seed treated with 3 mL/L Carrabiitol^®^ and foliar application at 2–3 leaf stage with 3 mL/L Carrabiitol^®^ + stress at flowering stage (T4), seed treated with 1 mL/L Carrabiitol^®^ and foliar application at pre-flowering stage (i.e., first emergence of flower bud) 1 mL/L Carrabiitol^®^ + stress at flowering stage (T5), seed treated with 2 mL/L Carrabiitol^®^ and foliar application at pre-flowering stage 2 mL/L Carrabiitol^®^ + stress at flowering stage (T6), and seed treated with 3 mL/L Carrabiitol^®^ and foliar application at pre-flowering stage with 3 mL/L Carrabiitol^®^ + stress at flowering stage (T7). Control plants were maintained in 3 groups: (i) absolute control—plants grown under normal (no abiotic stress) conditions and without Carrabiitol^®^; (ii) positive control—plants grown under normal conditions but with Carrabiitol^®^; and (iii) negative control—plants grown under different abiotic stress conditions and without Carrabiitol^®^. In order to induce abiotic stress, potted plants were exposed to salinity, flooding, heat, and drought, as detailed below. A total of four (4) replications were maintained for each treatment. The foliar spray of Carrabiitol^®^ was carried out at a rate of 150 L/acre. 

**Figure 1:**
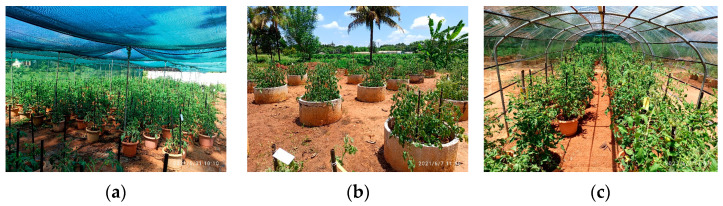
The illustrations depict the experimental setup for (**a**) control unstressed plants, (**b**) flooding stressed plants, and (**c**) salinity-stressed plants.

The flooding stress was imposed by submerging potted plants into a tank filled with water for 4 days at the flowering stage. Salinity treatment was imposed by irrigating plants with 50 mM NaCl (EC~4.6) for 8 days at the flowering stage (Figure 1a–c). 

**Figure 2:**
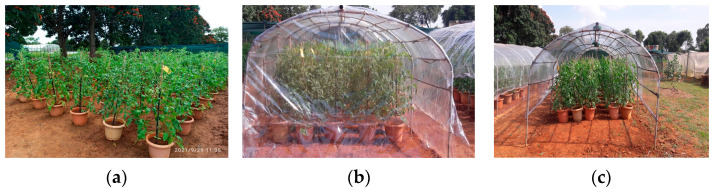
The illustrations depict the experimental setup for (**a**) control unstressed plants, (**b**) high-temperature stressed plants, and (**c**) drought-stressed plants.

Plants were exposed to a high temperature of 40 ± 1 °C. by keeping pots at the flowering stage in a polytunnel for 3 days. Control plants were kept outside under natural conditions of a day temperature of 29 ± 2 °C and a night temperature of 21 ± 2 °C. Drought was imposed by withholding water for 5 days at the flowering stage (Figure 2a–c). 

Carrabiitol^®^-treated plants exposed to salinity, flooding, high temperatures, and water deficits were evaluated for growth parameters, physiological characteristics, and biochemical contents after three (3) days of releasing the stress. 

### 2.2. Determination of Growth, Physiological, and Biochemical Parameters

#### 2.2.1. Flowering Days

Flowering time was determined by recording the number of days for the appearance of the first flower in plants after transplanting seedlings into pots. 

#### 2.2.2. Plant Dry Weight 

To determine dry weight, entire plants were gently uprooted and adhering soil removed in running tap water. Plants were kept between pads of blotting paper and dried in a hot air oven maintained at 80 °C for 48 h. The plant’s dry weight was recorded after cooling. 

#### 2.2.3. Gas Exchange Parameters

To determine leaf gas exchange, fully expanded healthy leaves (4th from top) were used for leaf gas exchange measurement using an open gas exchange system (Lichor 6400 XT, Lincoln, NE, USA). The system was calibrated prior to measurement. The gas exchange parameters of photosynthesis rate, stomatal conductance, and transpiration rate were determined between 10:00 and 11:30 h at 1200 μmol/m^2^/s photosynthetic photon flux density (PPFD), supplied by a red–blue LED light source built into the leaf chamber. The ambient CO_2_ concentration during measurement varied between 666 and 684 mg/m^3^. The leaf water potential was determined using a pressure bomb (Arimad-3000, MRC Ltd., Holon, Israel).

#### 2.2.4. Chrolophyll Content 

Leaf chlorophyll content was analyzed following the Hiscox and Israelstam methods [18]. A cork borer was used to cut out 10 mm leaf discs, and their weights were determined. The leaf discs were immersed in a 10 mL dimethyl sulfoxide (DMSO) solution and kept for 4 h in a hot air oven at 70 °C. The DMSO solution obtained was used to determine total chlorophyll content by recording absorbance at 645 and 663 nm in a UV–visible spectrophotometer (T80+ UV/VIS, PG Instruments Ltd., Lutterworth, UK). 

#### 2.2.5. Carotenoid Content 

The total carotenoid content was assessed using spectrophotometry. Carotenoids were extracted with acetone and partitioned with hexane to remove lipids. The carotenoids in the extract were estimated by reading the absorbance at 470 nm using a spectrophotometer (T80+ UV/VIS, PG Instruments Ltd., Lutterworth, UK).

#### 2.2.6. Total Antioxidant Activity Potential 

Total antioxidant activity potential was determined using 2,2-di phenyl-1-picryl hydrazyl (DPPH) radical assay [19]. A 0.2 mL aliquot of acidic methanol extract was mixed with 0.3 mL of 100 mM acetate buffer (pH 5.5) and 0.25 mL of ethanolic 0.5 mM DPPH solution. The reduction in color due to the scavenging of DPPH radicals by antioxidants was estimated by reading absorbance at 517 nm. Radical scavenging ability was expressed as IC50 values, where the weight of the sample required for a 50% reduction in DPPH radicals was calculated.

#### 2.2.7. Sugar Content 

The sugar (glucose and sucrose) compositions were determined by high-performance liquid chromatography (HPLC) (Prominence, Shimadzu, Kyoto, Japan) following de Cortes et al. [20]. The 2.0 g leaf samples were extracted in 20 mL of 70% methanol, and the contents were placed in a boiling water bath for 45 min. The extract was centrifuged at 5000 rpm at 25 °C, and the residue was re-extracted in another 10 mL of 80% ethanol and centrifuged again. All supernatants were pooled, dried in a rotary evaporator, and volume readjusted to 15 mL with distilled water. The samples were filtered using a 0.45 µm syringe filter (Millipore, Bedford, MA, USA) for the HPLC analysis. The HPLC system employed had a refractive index detector (Model 10A, Shimadzu) and an NH2 reversed-phase column (250 × 4.6 mm, 5 µm, Supelco, Bellefonte, PA, USA). During analysis, RID cell and column temperatures were maintained at 40 °C. The mobile phase consisted of water–acetonitrile (50:50, *v*/*v*) at a flow rate of 1.0 mL/min. The glucose and sucrose contents were quantified using a calibration curve prepared from their respective standards purchased from Sigma-Aldrich (Bangalore, India).

#### 2.2.8. MDA Content

The lipid peroxidation was estimated by determining the concentration of malondialdehyde (MDA) produced by the thiobarbituric acid (TBA) reaction [21]. Leaf material (1.0 g) was homogenized in 5 mL of 5% aqueous trichloroacetic acid (TCA) and 0.5 mL of 0.5% methanolic butylated hydroxytoluene (BHT) and heated for 30 min in boiling water. Then, the sample was centrifuged at 3000× *g* for 10 min. Then, to 1 mL of THE supernatant sample, 1 Ml of saturated TBA solution was added, and the contents were kept in a boiling water bath for 30 min. After centrifugation at 3000× *g* for 10 min, the absorbance of the solution was read at 532 to record the MDA level.

#### 2.2.9. Proline Content 

To determine proline content, the method of Bates [22] was used. The ground leaf sample (1.0 g) was mixed in 3% sulfosalicylic acid (aqueous), and the mix was centrifuged at 3000× *g*. The supernatant was mixed with 2 mL of ninhydrin and 2 mL of glacial acetic acid, and the solution was boiled for 1 h at 100 °C. The reaction was cooled to 25 ± 2 °C in an ice bath. After completion of the reaction, 4 mL toluene was added to the extract, and absorbance at 520 nm was read in a spectrophotometer. (T80+ UV/VIS, PG Instruments Ltd., Lutterworth, UK).

#### 2.2.10. Statistical Analysis

Data were subjected to a one-way analysis of variance (ANOVA). The means of four replicates were calculated for each treatment. Differences between treatment means were compared using Fisher’s post hoc least significant difference. The standard error of the mean (SEM) and LSD were reported for each ANOVA analysis. The results of all 7 treatments were used for principal component analysis (PCA) using OriginPro software (Origin 2022b, OriginLab Corporation, Northampton, MA, USA). Mean values were used to generate the correlation matrix, and the matrix comprised values of 12 traits in rows and 7 variables in columns. The cumulative variability, along with eigenvalues and principal component scores, were calculated, and a PCA biplot was developed.

## 3. Results

### 3.1. Effect of Carrabiitol Treatment on Growth Parameter 

#### 3.1.1. Flowering Days 

Since all treatments were imposed at the flowering stage, no data on the effect of Carrabiitol^®^ on the number of days for flowering from transplanting were determined. Data were taken only on Carrabiitol^®^ untreated (absolute control) and treated (positive control) plants (Table 1). 

In the positive control plants, the Carrabiitol^®^ treatments did not alter the number of days required for flowering after transplanting. The absolute control plants (T1) took longer flowering times compared to those treated with combinations of Carrabiitol^®^. The treatment T7 exhibited slightly early flowering.

#### 3.1.2. Plant Dry Weight

The plant’s dry weight was seen to decline significantly under flooding, drought, salinity, and high temperatures. Table 1 provides the data for plant dry weight in plants exposed to flooding and salinity. Carrabiitol^®^ treatment had a significant effect on the recovery of plant dry weight in plants exposed to salinity and flooding. The highest recovery of 91.45% was obtained in T7 treatment in plants exposed to flooding. Similarly, under salinity, increased plant dry weight was noted most effectively in treatments T6, T7, T4, and T2 (Table 1).

Table 2 provides the data for plant dry weight for plants exposed to heat and drought conditions. Under drought, the plant’s dry weight fell drastically by 44.83% compared to untreated plants (absolute control). The Carrabiitol^®^-treated plants showed a significant increase in dry weight under all the treatments, and the highest increase was found in treatment T6, followed by treatments T7, T4, and T5 under drought. Plants grown at high temperatures also showed a decline in plant dry weight and recovery under Carrabiitol^®^ treatments. However, the highest recovery of 22.70% was obtained in treatment T7 in the case of plants grown at high temperatures. The data are presented in Table 2 below. 

In the case of positive control plants, plant dry weight increased as the Carrabiitol^®^ dose was increased, and affectivity was most pronounced in treatment T7 by 76.28% during the July–September season of 2021. 

### 3.2. Effects of Carrabiitol^®^ Treatment on Physiological Parameters

#### 3.2.1. Leaf Water Potential (ψw)

Table 3 and Table 4 provide the leaf water potential (LWP) data for pants growing under (i) flooding and salinity and (ii) high temperature and drought conditions, respectively. The flooding and salinity considerably declined the ψw, and under both stressed conditions, Carrabiitol^®^ treatment showed increasing trends. The increase in ψw was high under T7 (13.04%) in flooding conditions and under T4 (18.75%) in salinity conditions as compared to T1 of the respective conditions. 

Under varied Carrabiitol^®^ treatments, the ψw ranged from −0.64 to −0.72 MPa in positive control plants, whereas it declined by 25.00% under high temperatures and by 17.24% under drought. The Carrabiitol^®^ treatments improved the ψw both in positive control and high-temperature or drought-stressed plants. The value for ψw was recorded highest in positive control, under T7 in high temperature, and under T6 in drought conditions (Table 4).

#### 3.2.2. Gas Exchange Parameters

Under flooding stress, photosynthesis rate, transpiration rate, and stomatal conductance declined by 46.50, 48.36, and 53.33% as compared to absolute control. (Table 3). Carrabiitol^®^ treatments showed marginal improvements in the values of these gas exchange variables in flooding and salinity-stressed plants over the control plants. The effect was pronounced under flooding in treatment T7. In the case of salinity, the Carrabiitol^®^ treatments influenced the photosynthesis rate, as evident in treatment T6; the effect on stomatal conductance and transpiration rate were more pronounced under treatments T7 and T4, respectively (Table 3). The gas exchange parameters registered a significant decline in the case of plants grown under high temperatures and drought conditions (Table 4). The Carrabiitol^®^ treatments significantly improved photosynthesis rate, stomatal conductance, and transpiration rate in both the positive control and stressed plants. The effect on the photosynthesis rate was radically significant under T7 (24.75%) and T6 (22.25%) in positive control plants in this study. 

### 3.3. Effects of Carrabiitol^®^ Treatment on Biochemical Parameters

#### 3.3.1. Chlorophyll Content

The flooding and salinity conditions led to a significant decline in chlorophyll content (Table 5). Treatments T4 and T3 were most effective in recovering the reduced chlorophyll content in flooding-stressed plants by 63.88 and 56.82%, respectively, whereas treatments T7, T4, and T3 (74.14, 61.46, and 58.04%, respectively) were found effective in salinity-stressed plants (Table 5). In positive control plants, treatment T4 increased chlorophyll content by 42.63%, followed by treatment T7. During high-temperature stress, Carrabiitol^®^ treatment T7 was found to be most effective, showing an increase in chlorophyll content of 35.47%, followed by treatments T6 and T5. In the drought-stressed plants, Carrabiitol^®^ did not show any recovery for chlorophyll reduction (Table 6). The research results showed that Carrabiitol^®^ application contributes to a higher synthesis of photosynthetic pigments in tomato leaves and thus improves plant survival under subsequent stress. It was also noted that Carrabiitol^®^ treatment was more effective when booster doses were applied at the 2–3 leaf stage instead of at the pre-flowering stage. 

#### 3.3.2. Total Carotenoid Content

The carotenoids play an important role in providing protection to membranes against oxidative stress. In the positive control plants, the Carrabiitol^®^ treatments T7 and T5 showed 15.08% and 17.24% carotenoid increases, respectively, over plants raised from untreated seeds. The data for the same are presented in Table 5. Flooding and salinity conditions led to a decline in carotenoid content of 41.81 and 22.84%, respectively; however, under Carrabiitol^®^ treatment, a reversal in the decline of carotenoid content was observed. Treatment T7 plants recovered carotenoid content by 30.73% in flooding stress-induced decline. In the case of plants grown under salinity conditions, treatments T4, T7, and T5 exhibited a positive influence of 12.29, 10.61, and 10.05%, respectively. However, in high-temperature or drought-stressed plants, the Carrabiitol^®^ treatments marginally controlled the decreased content of carotenoids. The data are presented in Table 6. Under high-temperature conditions, the Carrabiitol^®^ treatment T3 exhibited a 19.10% recovery, and treatment T5 showed a 13.48% recovery. In the case of plants grown under drought conditions, treatments T6, T4, and T7 were found effective, with a 21.27% and 18.72% recovery, respectively. In this study, a dose-dependent improvement was seen in the carotenoid content of plants after Carrabiitol^®^ treatment at the 2–3 leaf stage as well as the pre-flowering stage. The results indicate that Carrabiitol^®^ treatments prevent pigment degradation due to stressed conditions.

#### 3.3.3. Total Antioxidant Activity Potential

The DPPH assay is considered an important determinant of free radical scavenging capacity. The antioxidant activity potential in the untreated or Carrabiitol^®^-treated and stressed plants did not show significant differences under the flooding and salinity conditions. The data for the same are presented in Table 5. It was observed that under both stressed conditions, plants led to a considerable decline in antioxidant activity potential, and the same was not recovered in the stressed plants treated by the Carrabiitol^®^ treatments. The antioxidant activity potential of the negative control plants decreased under high temperatures and drought conditions compared to the absolute control plants. The Carrabiitol^®^ treatments increased antioxidant activity potential prominently under the T4 (40.00%) and T6 (31.80%) treatments in the plants exposed to high temperatures and under T7 (52.25%) and T6 (35.58%) in the drought-exposed plants over the negative control. The data are presented in Table 6.

#### 3.3.4. Sugar Content

Sugars, namely glucose and sucrose, are important osmotically active biomolecules playing roles in many physiological events, and thus, their alteration due to stressed conditions in plants has relevance in the adaptation of these stresses. The Carrabiitol^®^ treatments in the positive control plants showed a significant increase in glucose (21.71–56.86%) and sucrose content by 12.13–67.78%. Data are presented in Table 5 and Table 7 for glucose and sucrose, respectively, for plants grown under flooding and salinity conditions. It was observed that the flooding and salinity led to a decline in glucose and sucrose contents, and Carrabiitol^®^ treatment recovered these declines. An increase in glucose content of 23.98–56.81% was detected in the flooding-stressed plants; a 19.00–56.38% increase was noted in salinity-stressed plants. Carrabiitol^®^ treatments T7 and T6 were effective in regulating glucose content in the flood-stressed plants, whereas T6 treatment was found effective for similar regulation of glucose content in the salinity-stressed plants.

Similarly, the Carrabiitol^®^ treatment in the flooding and salinity-stressed plants showed an increase in sucrose content of 9.69–34.69% and 7.09–89.67% in comparison to the negative control. The effect was prominent in T6 (34.69%) and T7 (30.10%) treatments in plants grown under flooding conditions. This effect was reflected in T7 (89.67%), T3 (40.64%), and T6 (33.54%) treatments in the case of plants grown under salinity conditions. The high temperature and drought decreased the glucose and sucrose contents in the negative control plants. The data for the decline in glucose and sucrose for plants grown under high temperatures and drought conditions are presented in Table 6 and Table 8, respectively. Under different Carrabiitol^®^ treatments, glucose and sucrose contents increased in the positive control plants and also controlled the observed decline in glucose and sucrose contents in the stressed plants significantly. The effect was marked with respect to glucose content under T7 and T6 by 46.34% and 41.01%, respectively, in the high-temperature stressed plant; whereas, in the case of drought-stressed plants, treatment T5 detected 65.73% and treatment T7 led to 62.30% glucose content. In the case of high-temperature and drought-stressed plants, treatment T7 was found to be most effective in overcoming the decline in sucrose content. 

#### 3.3.5. Malondialdehyde Content

In the present study, the MDA production under flooding and salinity led to an increase of 94.82 and 70.68%, respectively. The data are presented in Table 7 Treatments with Carrabiitol^®^ demonstrated regulatory responses to MDA content in plants affected by flooding (23.89–46.01%) and salinity (17.17–39.39%). The effect of Carrabiitol^®^ treatment was high under T4 (46.01%), T7 (41.59%), and T5 (36.28%) in the flooding-stressed plants, whereas T4 (39.39%), T6 (31.31%), and T7 (29.29%) treatments were found effective in the salinity-stressed plants. The high temperature and drought led to a significant surge in lipid peroxidation. However, the Carrabiitol^®^ treatments balance the content of lipid peroxidation. Such an effect of Carrabiitol^®^ was prominent in T7 and T6 treatments under both stressed conditions. The data are presented in Table 8. The foliar application at the pre-flowering stage was comparatively more pronounced than at the 2–3 leaf stage. 

#### 3.3.6. Proline Content

The flooding and salinity in the present study significantly increased proline content. The Carrabiitol^®^ treatments exhibited an up-regulatory response to proline content both in positive control and in flooding or salinity-stressed plants. In the positive control plants, the proline content under the Carrabiitol^®^ treatments was increased by 10 to 100-fold as compared to absolute control plants, and the effect was drastic under T7, T6, and T3. The data are presented in Table 7.

Similarly, after receiving treatments with Carrabiitol^®^, the proline concentration increased in the plants exposed to flooding and salinity stress by 100 to 200 times and 10 to 100 times, respectively. The effect was pronounced in the flooding-stressed plants under T7 and T6 treatments, whereas in the salinity-stressed plants under T6 and T3 treatments. In a similar manner, the high temperature and drought led to an increase in proline, which was upregulated by the Carrabiitol^®^ treatments under both stressed conditions. As suggested from the data represented in Table 8, the treatments T6 (45.53%) and T4 (31.18%) were the most effective in the high-temperature stressed plants, whereas T5 (52.73%), T7 (47.69%), and T4 (46.40%) in the drought-stressed plants. 

### 3.4. Statistical Analysis

**Figure 3:**
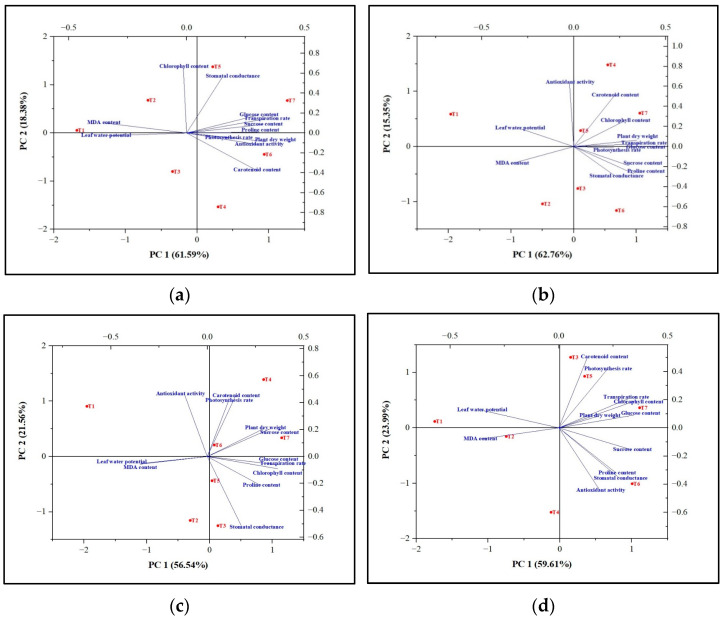
Principal component analysis (PCA) biplots showing the tolerance capacity of tomato plants toward the abiotic stresses viz. (**a**) Drought, (**b**) Flooding, (**c**) Salinity, and (**d**) High tempreature. T1 = without Carrabiitol^®^; T2, T3, and T4 = seed treatment and foliar application at 2–3 leaf stage with 1 mL/L, 2 mL/L, and 3 mL/L Carrabiitol^®^, respectively, + stress at flowering stage; T5, T6, and T7 = seed treatment and foliar application at pre-flowering stage with 1 mL/L, 2 mL/L, and 3 mL/L Carrabiitol^®^, respectively, + stress at flowering stage. Vectors pointing in similar directions indicate positively correlated variables; vectors pointing in opposite directions indicate negatively correlated variables; and vectors pointing in the right direction indicate a lack of correlation. The vector pointing toward the particular treatment indicates the positive or negative correlation toward that treatment based on the occurrence of the treatment code in each quadrant. The variability of the variable represented by the two principal components increases with the length of the vectors.

The data from all seven treatments were also used to assess the tolerance capacity of tomato plants to abiotic stress by employing PCA analysis (Figure 3). The principal components 1 (PC1) and 2 (PC2) had eigenvalues greater than 1 (Table 9). The cumulative variabilities were found to be 78.10%, 78.11%, 83.60%, and 79.97% for induced flooding, salinity, high temperature, and drought conditions, respectively (Figure 1 and Table 9). 

The results of the PCA analysis of flooding indicated that Carrabiitol^®^ treatment was effective against the induced flooding stress as compared to untreated plants (T1). Treatment T7 was found to be the most significant treatment against the induced flooding stress, followed by T5 and T4 treatments in maintaining the plant’s dry weight, chlorophyll content, transpiration rate, photosynthesis rate, proline content, glucose content, and sucrose content. Treatment T2 was not effective against the induced flooding stress (Figure 3b). The results of the PCA analysis of salinity indicated that Carrabiitol^®^ treatment was effective against the induced salinity stress as compared to untreated plants (T1). Treatment T7 was the most significant treatment against the induced salinity stress, followed by T6 and T4 treatments in maintaining the plant’s dry weight, sucrose content, chlorophyll content, transpiration rate, proline content, and glucose content. Treatments T2 was not effective against induced salinity stress (Figure 3c). The results of the PCA analysis of high temperatures indicated that Carrabiitol^®^ treatment was effective against the induced high-temperature stress as compared to untreated plants (T1). Treatment T7 was the most significant treatment against the induced high-temperature stress in maintaining the plant dry weight, chlorophyll content, transpiration rate, and glucose content, whereas treatment T6 was the most significant treatment against the induced high-temperature stress in maintaining the stomatal conductance, proline content, sucrose content, and antioxidant activity potential. Other treatments have a milder effect against high-temperature stress (Figure 3d). The results of the PCA analysis of drought indicated that Carrabiitol^®^ treatment was effective against the induced drought stress as compared to untreated plants (T1). Treatment T7 was the most significant treatment against the induced drought stress, followed by T5 and T6 treatments for maintaining the plant’s dry weight, transpiration rate, photosynthesis rate, proline content, glucose content, sucrose content, and antioxidant activity potential. Treatments T3 and T2 were not effective against drought-induced stress (Figure 3a). Overall, treatment T7 was the most significant treatment under which tomato plants were able to sustain themselves against induced stress conditions.

## 4. Discussion

In the present study, the effect of Carrabiitol^®^ was assessed on tomato plants grown under different stress conditions. This study was carried out in the open field of the ICAR-IIHR research farm located in Bengaluru, India. The research work was conducted during two cropping seasons. 

Biomass reallocation represents an important plasticity trait in plant responses to environmental stress because of the specific functionalities and metabolic costs of different plant tissues and organs. In order to maximize chances for growth and survival, plants must optimize their biomass allocation depending on the specific surrounding environment [23]. In the present study, plants treated with Carrabiitol^®^ showed higher plant biomass in both stressed and unstressed conditions. This indicates that Carrabiitol^®^ treatment is effective in promoting increased plant growth and also acts as a protector against stress.

Leaf water potential (LWP) is an indicator of plant water status [24,25]. The results of this study reveal that plants treated with Carrabiitol^®^ were less sensitive to water deprivation compared to untreated plants. It is thus evident that Carrabiitol^®^ treatment helps to maintain homeostasis in plant cells under stressed conditions.

Stomatal opening regulates the important physiological parameters of the plant, namely stomatal conductance, transpiration rate, and photosynthesis rate. The photosynthetic capacity of leaves directly determines the level of plant productivity [26]. Carrabiitol^®^ treatments showed marginal improvement in the values of the above-mentioned gas exchange variables even under stressed conditions. Such improvements in the gas exchange variables may accelerate the accumulation of nutrients in plants, thereby promoting the maturation and increasing the yield of tomatoes [27]. For instance, plants exposed to high temperatures showed a decline in transpiration rate of 35.52% as compared to unstressed plants. Carrabiitol^®^ treatment helped to reduce this decline by a significant margin. It was observed that a Carrabiitol^®^ concentration of 3 mL/L was most effective, irrespective of its application at the 2–3 leaf stage or pre-flowering stage.

The production of free radicals during abiotic stress is deleterious for plant survival, and thus, regulation of free radical production is important for plant survival under stressed conditions. Malondialdehyde is commonly associated with the induced peroxidation of cellular polyunsaturated fatty acids under stressed conditions, and its levels serve as a marker of oxidative stress created by varied stress. The results of this study indicated that the application of Carrabiitol^®^ led to decreased MDA content in tomato plants subjected to abiotic stress conditions. Lipid peroxidation results from an increase in ROS, such as superoxide radical (O_2_•^−^), hydrogen peroxide (H_2_O_2_), and hydroxyl radical (OH•) in chloroplasts [28]. The treatments with Carrabiitol^®^ decreased the MDA level in the leaves of stressed plants, indicating there may have been a lower accumulation of ROS in the plants.

An increase in the concentration of soluble sugars has been reported to enhance plant tolerance to several abiotic stresses, such as drought, salinity, and cold [29]. In fact, in developing plant embryos, glucose and sucrose have been reported to regulate cell division, cell expansion, and the accumulation of reserve carbohydrates [30,31,32]. Proline, on the other hand, is an amino acid that helps plants protect themselves from abiotic stress. It acts through its regulatory actions on stabilization of sub-cellular structures, scavenging of free radicals, and buffering cellular redox potential. Proline has also been found to be important for the stability and protection of enzymes, proteins, and membranes from drought-induced osmotic stress [33].

In the present study, the proline content was found to increase in a dose-dependent manner at all stages of Carrabiitol^®^ application. These results indicate that increased proline content protects cells from water loss and maintains an osmotic balance that may protect enzymes and stabilize macromolecules.

Under abiotic conditions, the increased concentration of reactive oxygen species (ROS) has been known to cause oxidative stress that damages the macromolecule structures in plant cells. In order to minimize the effect of ROS stress, plants release osmoprotectants like sucrose, glucose, and proline, which protect macromolecules and prevent damage. Carrabiitol^®^ treatment showed an increase in this molecular concentration, thus establishing positive and protective effects on tomato plants under induced abiotic stress conditions.

The results of the present research study go a long way toward corroborating the hypothesis that Carrabiitol^®^ can act as a compatible solute. The results of this study have shown improvement in plant water status and a positive effect on normalizing all the physiological processes like photosynthesis rate, transpiration rate, and stomatal conductance under stressed conditions. The results for chlorophyll and carotenoid content indicate that Carrabiitol^®^ treatment can protect the photosystem apparatus under detrimentally stressed conditions. 

## 5. Conclusions

The Carraiitol^®^ formulation was found to influence the physiological parameters in the tomato plants grown under stress conditions. Seed priming, along with booster dosages at the 2–3 leaf stage and pre-flowering stages, were found to be most effective in relieving abiotic stress. The findings indicate that Carrabiitol^®^ can be an effective agricultural stimulant, promoting plant growth under different abiotic stress conditions. However, more studies on the effects of treatment on yield and quality attributes under different stress conditions are required to substantiate and confirm the stress alleviation potential of the Carrabiitol^®^ formulation.

## 6. Patents

1. IN396252: Shah, N.J.; Patel, F.Y., Carrabiitol formulation to maintain osmotic balance in plants against abiotic stress and method of extraction & preparation thereof, **2022** Applicant: M/s Pushpa J Shah.
2. WO2022064524A1: Shah, N.J.; Patel, F.Y., Carrabiitol formulation to maintain osmotic balance in plants against abiotic stress and method of extraction & preparation thereof’, **2022** Applicant: M/s Pushpa J Shah.

## Figures and Tables

**Table 1:** Effects of flooding and salinity on flowering time and plant dry weight of tomato plants after treatments with Carrabiitol^®^.

Treatment	Flowering Time (Days)	Plant Dry Weight (g)		
		Positive Control	Flooding	Salinity
T1	29.50	49.15 ^a^	29.03 ^a nc^	36.44 ^a nc^
T2	29.75 ^a^	56.07 ^ab^	42.40 ^b^	40.98 ^ab^
T3	28.25 ^a^	59.47 ^ab^	41.90 ^b^	38.91 ^a^
T4	28.00	62.08 ^ab^	47.95 ^bc^	46.23 ^ab^
T5	28.25 ^a^	56.58 ^ab^	50.68 ^bc^	38.15 ^a^
T6	27.50 ^a^	58.83 ^ab^	48.18 ^bc^	39.75 ^ab^
T7	27.38 ^a^	65.17 ^b^	55.58 ^c^	50.35 ^b^
SEM	0.35	1.92	3.22	1.92
F-value	0.34	1.13	5.79	1.78
LSD (*p* = 0.05)	NS	14.07	10.40	10.94

^nc^ = negative control; T1 = without Carrabiitol^®^; T2, T3, and T4 = seed treatment and foliar application at 2–3 leaf stage with 1 mL/L, 2 mL/L, and 3 mL/L Carrabiitol^®^, respectively, + stress at flowering stage; T5, T6, and T7 = seed treatment and foliar application at pre-flowering stage with 1 mL/L, 2 mL/L, and 3 mL/L Carrabiitol^®^, respectively, + stress at flowering stage. Means followed by a common letter are not significantly different (LSD test, *p* < 0.05). NS—not significant.

**Table 2:** Effect of high temperature and drought on flowering time and plant dry weight of tomato plants after treatment with Carrabiitol^®^.

Treatment	Flowering Time (Days)	Plant Dry Weight (g)		
		Positive Control	High Temperature	Drought
T1	28.25 ^a^	42.89 ^a nc^	29.86 ^a nc^	23.66 ^a nc^
T2	29.25 ^a^	63.25 ^ab^	33.62 ^a^	40.18 ^b^
T3	29.25 ^a^	62.59 ^ab^	30.55 ^a^	36.68 ^b^
T4	28.75 ^a^	56.08 ^ab^	32.91 ^a^	46.68 ^c^
T5	29.25 ^a^	69.81 ^b^	35.61 ^a^	40.33 ^b^
T6	27.75 ^a^	62.15 ^ab^	31.09 ^a^	48.08 ^c^
T7	27.5 ^a^	75.61 ^b^	36.64 ^a^	47.24 ^c^
SEM	0.28	3.93	0.98	3.23
F-value	0.56	2.20	1.08	18.37
LSD (*p* = 0.05)	NS	20.62	NS	5.87

^nc^ = negative control; T1 = without Carrabiitol^®^; T2, T3, and T4 = seed treatment and foliar application at 2–3 leaf stage with 1 mL/L, 2 mL/L, and 3 mL/L Carrabiitol^®^, respectively, + stress at flowering stage; T5, T6, and T7 = seed treatment and foliar application at pre-flowering stage with 1 mL/L, 2 mL/L, and 3 mL/L Carrabiitol^®^, respectively, + stress at flowering stage. Means followed by a common letter are not significantly different (LSD test, *p* < 0.05). NS—not significant.

**Table 3:** Effects of flooding and salinity on physiological parameters of tomato plants after treatments with Carrabiitol^®^.

	Leaf Water Potential (-MPa)			Photosynthesis Rate (μmol/m^2^/s)			Stomatal Conductance (mmol/m^2^/s)			Transpiration Rate (mmol/m^2^/s)		
Treatment	Positive Control	Flooding	Salinity	Positive Control	Flooding	Salinity	Positive Control	Flooding	Salinity	Positive Control	Flooding	Salinity
T1	0.83 ^a^	1.15 ^a nc^	1.28 ^anc^	15.02 ^ab^	8.04 ^a nc^	9.53 ^ab nc^	0.15 ^a^	0.07 ^a nc^	0.05 ^anc^	3.97 ^a^	2.05 ^e nc^	2.01 ^anc^
T2	0.80 ^a^	1.01 ^b^	1.10 ^bc^	13.49 ^a^	8.28 ^ab^	8.99 ^ab^	0.12 ^a^	0.08 ^ab^	0.08 ^b^	4.17 ^a^	3.21 ^a^	2.88 ^b^
T3	0.78 ^ab^	1.03 ^b^	1.16 ^b^	17.59 ^b^	7.92 ^a^	8.83 ^b^	0.13 ^a^	0.10 ^c^	0.08 ^b^	5.00 ^b^	3.47 ^ab^	2.88 ^b^
T4	0.77 ^ab^	1.01 ^b^	1.04 ^c^	16.23 ^ab^	9.03 ^ab^	10.41 ^ab^	0.14 ^a^	0.07 ^a^	0.06 ^ac^	5.82 ^c^	4.01 ^bc^	3.25 ^b^
T5	0.82 ^a^	1.09 ^c^	1.16 ^b^	14.65 ^ab^	9.24 ^ab^	9.10 ^ab^	0.12 ^a^	0.09 ^bc^	0.07 ^bc^	4.31 ^a^	3.55 ^abc^	3.10 ^b^
T6	0.78 ^ab^	1.02 ^b^	1.14 ^bd^	13.90 ^a^	9.90 ^b^	11.15 ^a^	0.15 ^a^	0.09 ^bc^	0.06 ^ac^	5.64 ^cd^	4.16 ^cd^	2.85 ^b^
T7	0.73 ^b^	1.00 ^b^	1.07 ^cd^	14.40 ^ab^	8.73 ^ab^	9.99 ^ab^	0.14 ^a^	0.10 ^c^	0.08 ^b^	5.27 ^bd^	4.69 ^d^	3.24 ^b^
SEM	0.01	0.02	0.03	0.54	0.27	0.32	0.00	0.00	0.00	0.28	0.32	0.16
F-value	2.00	11.71	8.31	1.50	1.56	1.23	1.84	3.88	3.18	17.01	15.01	5.24
LSD (*p* = 0.05)	0.07	0.05	0.08	3.42	1.68	2.26	NS	0.01	0.01	0.53	0.64	0.54

^nc^ = negative control; T1 = without Carrabiitol^®^; T2, T3, and T4 = seed treatment and foliar application at 2–3 leaf stage with 1 mL/L, 2 mL/L, and 3 mL/L Carrabiitol^®^, respectively, + stress at flowering stage; T5, T6, and T7 = seed treatment and foliar application at pre-flowering stage with 1 mL/L, 2 mL/L, and 3 mL/L Carrabiitol^®^, respectively, + stress at flowering stage. Means followed by a common letter are not significantly different (LSD test, *p* < 0.05). NS—not significant.

**Table 4:** Effects of high temperature and drought on physiological parameters of tomato plants after treatments with Carrabiitol^®^.

	Physiological Analysis											
	Leaf Water Potential (-MPa)			Photosynthesis Rate (μmol/m^2^/s)			Stomatal Conductance (mmol/m^2^/s)			Transpiration Rate (mmol/m^2^/s)		
Treatment	Positive Control	High Temperature	Drought	Positive Control	High Temperature	Drought	Positive Control	High Temperature	Drought	Positive Control	High Temperature	Drought
T1	0.72 ^a^	0.90 ^a nc^	0.87 ^a nc^	12.04 ^ac^	7.17 ^ab nc^	8.70 ^ac nc^	0.14 ^a^	0.07 ^a nc^	0.10 ^abc nc^	2.86 ^a^	1.84 ^a nc^	1.57 ^a nc^
T2	0.68 ^abc^	0.83 ^b^	0.82 ^b^	13.26 ^ab^	6.98 ^a^	7.95 ^ab^	0.16 ^ab^	0.09 ^ab^	0.11 ^abc^	3.40 ^abc^	1.80 ^a^	2.32 ^b^
T3	0.67 ^bc^	0.80 ^bc^	0.80 ^bc^	12.26 ^ac^	9.01 ^d^	7.66 ^b^	0.18 ^ab^	0.08 ^ab^	0.09 ^ac^	3.62 ^bc^	2.40 ^bd^	1.98 ^ab^
T4	0.69 ^ab^	0.77 ^c^	0.81 ^bc^	11.33 ^c^	6.40 ^a^	8.46 ^abc^	0.20 ^ab^	0.10 ^b^	0.08 ^a^	3.11 ^ab^	2.04 ^abd^	2.21 ^b^
T5	0.71 ^ab^	0.78 ^bc^	0.80 ^bc^	11.21 ^c^	8.02 ^bc^	8.16 ^abc^	0.19 ^ab^	0.09 ^ab^	0.12 ^bc^	4.01 ^c^	2.89 ^c^	2.34 ^b^
T6	0.67 ^bc^	0.75 ^c^	0.77 ^c^	14.72 ^bd^	7.98 ^bc^	9.77 ^d^	0.17 ^ab^	0.10 ^b^	0.11 ^abc^	3.70 ^bc^	2.61 ^cd^	2.88 ^c^
T7	0.64 ^c^	0.75 ^c^	0.78 b^c^	15.02 ^d^	8.86 ^cd^	8.98 ^cd^	0.22 ^b^	0.10 ^b^	0.13 ^b^	3.89 ^bc^	3.03 ^c^	3.01 ^c^
SEM	0.01	0.02	0.01	0.59	0.37	0.27	0.01	0.00	0.01	0.16	0.19	0.19
F-value	3.82	9.22	5.27	7.96	9.69	4.63	1.81	2.79	4.16	3.71	10.41	8.23
LSD (*p* = 0.05)	0.04	0.05	0.04	1.62	0.92	0.97	0.06	0.02	0.03	0.64	0.45	0.51

^nc^ = negative control; T1 = without Carrabiitol^®^; T2, T3, and T4 = seed treatment and foliar application at 2–3 leaf stage with 1 mL/L, 2 mL/L, and 3 mL/L Carrabiitol^®^, respectively, + stress at flowering stage; T5, T6, and T7 = seed treatment and foliar application at pre-flowering stage with 1 mL/L, 2 mL/L, 3 mL/L Carrabiitol^®^, respectively, + stress at flowering stage. Means followed by a common letter are not significantly different (LSD test, *p* < 0.05). NS—not significant.

**Table 5:** Effects of flooding and salinity on photochemical & antioxidant parameters of tomato plants after treatments with Carrabiitol^®^.

	Biochemical Analysis											
	Chlorophyll Content (mg/g FW)			Carotenoid Content (mg/g FW)			Total Antioxidant Activity Potential (DPPH, mg/100 g)			Glucose Content (mg/g FW)		
Treatment	Positive Control	Flooding	Salinity	Positive Control	Flooding	Salinity	Positive Control	Flooding	Salinity	Positive Control	Flooding	Salinity
T1	3.26 ^a^	2.27 ^a nc^	2.05 ^e nc^	2.32 ^a^	1.35 ^a nc^	1.79 ^ae nc^	121.59 ^a^	54.13 ^a nc^	71.28 ^a nc^	6.77 ^a^	3.96 ^a nc^	3.21 ^a nc^
T2	3.61 ^a^	2.53 ^ac^	2.84 ^ab^	1.81 ^b^	1.09 ^b^	1.41 ^b^	94.27 ^b^	44.64 ^b^	46.31 ^b^	8.79 ^b^	5.18 ^bd^	2.99 ^b^
T3	4.18 ^b^	3.56 ^b^	3.24 ^bcd^	2.17 ^a^	1.41 ^a^	1.59 ^bd^	75.52 ^c^	39.99 ^c^	49.92 ^c^	8.24 ^b^	5.32 ^b^	3.84 ^c^
T4	4.65 ^b^	3.72 ^b^	3.31 ^cd^	2.67 ^cd^	1.71 ^c^	2.01 ^c^	124.99 ^a^	59.35 ^d^	69.85 ^a^	10.62 ^c^	5.61 ^c^	4.21 ^d^
T5	4.27 ^b^	3.40 ^b^	3.05 ^ac^	2.72 ^c^	1.63 ^c^	1.97 ^ac^	82.31 ^d^	42.09 ^bc^	44.64 ^bd^	9.27 ^b^	4.91 ^d^	3.82 ^c^
T6	3.52 ^a^	2.92 ^c^	2.71 ^a^	2.40 ^ad^	1.45 ^a^	1.76 ^de^	62.85 ^e^	39.60 ^c^	41.78 ^d^	10.49 ^c^	5.86 ^c^	5.02 ^e^
T7	4.29 ^b^	3.41 ^b^	3.57 ^d^	2.67 ^cd^	1.76 ^c^	1.98 ^ac^	86.24 ^d^	55.53 ^ad^	52.00 ^e^	8.28 ^b^	6.21 ^e^	3.98 ^f^
SEM	0.19	0.21	0.19	0.13	0.09	0.09	8.75	3.10	4.54	0.51	0.28	0.25
F-value	7.34	13.88	11.02	11.63	15.77	13.36	119.88	37.40	162.46	13.86	67.53	281.57
LSD (*p* = 0.05)	0.55	0.43	0.44	0.29	0.18	0.19	6.41	4.07	2.86	1.10	0.27	0.12

^nc^ = negative control; T1 = without Carrabiitol^®^; T2, T3, and T4 = seed treatment and foliar application at 2–3 leaf stage with 1 mL/L, 2 mL/L, and 3 mL/L Carrabiitol^®^, respectively, + stress at flowering stage; T5, T6, and T7 = seed treatment and foliar application at pre-flowering stage with 1 mL/L, 2 mL/L, and 3 mL/L Carrabiitol^®^, respectively, + stress at flowering stage. Means followed by a common letter are not significantly different (LSD test, *p* < 0.05). NS—not significant.

**Table 6:** Effects of high temperature and drought on photochemical & antioxidant parameters of tomato plants after treatments with Carrabiitol^®^.

	Biochemical Analysis											
	Chlorophyll Content (mg/g FW)			Carotenoid Content (mg/g FW)			Total Antioxidant Activity Potential (DPPH, mg/100 g)			Glucose Content (mg/g FW)		
Treatment	Positive Control	High Temperature	Drought	Positive Control	High Temperature	Drought	Positive Control	High Temperature	Drought	Positive Control	High Temperature	Drought
T1	4.50 ^a^	2.96 ^a nc^	3.32 ^ab nc^	3.54 ^a^	1.78 ^a nc^	2.35 ^a nc^	93.02 ^a^	41.32 ^ad nc^	37.85 ^a nc^	5.48 ^a^	3.56 ^a nc^	3.21 ^a nc^
T2	3.85 ^b^	3.32 ^ab^	3.51 ^b^	3.55 ^a^	1.80 ^ab^	2.41 ^a^	57.85 ^b^	38.39 ^a^	36.96 ^a^	7.17 ^bc^	4.36 ^b^	3.83 ^b^
T3	4.65 ^ac^	3.75 ^cd^	3.18 ^ab^	3.02 ^bd^	2.12 ^b^	2.73 ^bc^	77.67 ^ce^	38.92 ^a^	40.35 ^a^	7.56 ^bcd^	4.79 ^c^	4.72 ^c^
T4	3.81 ^b^	3.40 ^bd^	3.05 ^a^	3.41 ^ae^	1.76 ^a^	2.79 ^bc^	63.56 ^bd^	57.85 ^b^	46.42 ^b^	6.75 ^b^	4.12 ^d^	4.22 ^d^
T5	4.76 ^ac^	3.96 ^c^	3.56 ^b^	3.37 ^ae^	2.02 ^ab^	2.48 ^ac^	69.81 ^cd^	44.46 ^d^	37.85 ^a^	8.12 ^cd^	4.42 ^b^	5.32 ^e^
T6	4.36 ^a^	3.80 ^cd^	3.21 ^ab^	2.76 ^bc^	1.80 ^ab^	2.85 ^b^	87.49 ^ae^	54.46 ^b^	51.32 ^c^	7.89 ^cd^	5.02 ^e^	4.75 ^c^
T7	5.02 ^c^	4.01 ^c^	3.45 ^ab^	3.19 ^de^	1.91 ^a^	2.79 ^bc^	76.96 ^ce^	49.74 ^c^	57.63 ^d^	8.39 ^d^	5.21 ^e^	5.21 ^e^
SEM	0.17	0.15	0.07	0.11	0.05	0.08	4.74	2.92	3.02	0.37	0.21	0.29
F-value	8.01	7.11	1.94	9.20	1.68	4.15	12.25	29.15	32.99	7.49	61.25	80.11
LSD (*p* = 0.05)	0.47	0.42	0.40	0.29	0.32	0.31	10.86	4.33	4.22	1.10	0.22	0.26

^nc^ = negative control; T1 = without Carrabiitol^®^; T2, T3, and T4 = seed treatment and foliar application at 2–3 leaf stage with 1 mL/L, 2 mL/L, and 3 mL/L Carrabiitol^®^, respectively, + stress at flowering stage; T5, T6, and T7 = seed treatment and foliar application at pre-flowering stage with 1 mL/L, 2 mL/L, and 3 mL/L Carrabiitol^®^, respectively, + stress at flowering stage. Means followed by a common letter are not significantly different (LSD test, *p* < 0.05). NS—not significant.

**Table 7:** Effects of flooding and salinity on biochemical parameters of tomato plants after treatments with Carrabiitol^®^.

	Biochemical Analysis								
	Sucrose Content (mg/g FW)			MDA Content (µg/g FW)			Proline (mg/100 g FW)		
Treatment	Positive Control	Flooding	Salinity	Positive Control	Flooding	Salinity	Positive Control	Flooding	Salinity
T1	2.39 ^a^	1.96 ^a nc^	1.55 ^a nc^	0.58 ^a^	1.13 ^a nc^	0.99 ^a nc^	2.29 ^a^	4.14 ^a nc^	6.53 ^a nc^
T2	3.15 ^bd^	2.45 ab	1.66 ^a^	0.44 ^b^	0.86 ^b^	0.82 ^ac^	3.46 ^b^	7.36 ^b^	8.15 ^b^
T3	2.68 ^ad^	2.49 ^ab^	2.18 ^b^	0.43 ^be^	0.79 ^bd^	0.73 ^bc^	4.87 ^c^	8.52 ^c^	10.82 ^cf^
T4	3.92 ^c^	2.57 ^b^	2.63 ^c^	0.36 ^c^	0.61 ^c^	0.60 ^b^	3.64 ^d^	8.06 ^d^	8.91 ^de^
T5	2.54 ^a^	2.15 ^ab^	1.86 ^ab^	0.38 ^d^	0.72 ^bc^	0.72 ^bc^	3.76 ^e^	8.62 ^e^	9.06 ^e^
T6	3.54 ^bc^	2.64 ^b^	2.07 ^b^	0.42 ^ef^	0.79 ^bd^	0.68 ^bc^	5.66 ^f^	10.88 ^f^	10.50 ^f^
T7	4.01 ^c^	2.55 ^b^	2.94 ^c^	0.41 ^f^	0.66 ^cd^	0.70 ^bc^	4.35 ^g^	8.91 ^g^	9.91 ^g^
SEM	0.25	0.09	0.19	0.03	0.06	0.05	0.41	0.77	0.56
F-value	12.26	1.72	19.33	3.07	10.80	3.51	78.63	213.69	128.85
LSD (*p* = 0.05)	0.58	0.58	0.35	0.01	0.16	0.20	0.03	0.42	0.40

MDA, malondialdehyde; ^nc^ = negative control; T1 = without Carrabiitol^®^; T2, T3, and T4 = seed treatment and foliar application at 2–3 leaf stage with 1 mL/L, 2 mL/L, and 3 mL/L Carrabiitol^®^, respectively, + stress at flowering stage; T5, T6, and T7 = seed treatment and foliar application at pre-flowering stage with 1 mL/L, 2 mL/L, and 3 mL/L Carrabiitol^®^, respectively, + stress at flowering stage. Means followed by a common letter are not significantly different (LSD test, *p* < 0.05). NS—not significant.

**Table 8:** Effects of high temperature and drought on biochemical parameters of tomato plants after treatments with Carrabiitol^®^.

	Biochemical Analysis								
	Sucrose Content (mg/g FW)			MDA Content (µg/g FW)			Proline (mg/100 g FW)		
Treatment	Positive Control	High Temperature	Drought	Positive Control	High Temperature	Drought	Positive Control	High Temperature	Drought
T1	2.97 ^a^	1.89 ^a nc^	1.56 a ^nc^	0.45 ^ab^	0.86 ^a nc^	0.76 a ^nc^	4.13 ^a^	8.85 ^a nc^	9.31 ^a nc^
T2	3.43 ^a^	2.17 ^ab^	1.76 ^ac^	0.52 ^ab^	0.79 ^ab^	0.62 ^abc^	4.80 ^b^	9.74 ^b^	11.15 ^b^
T3	3.35 ^a^	2.33 ^ab^	1.88 ^ac^	0.48 ^ab^	0.66 ^ab^	0.64 ^ab^	4.08 ^c^	10.83 ^cd^	12.68 ^c^
T4	4.25 ^b^	2.28 ^ab^	2.39 ^b^	0.53 ^a^	0.73 ^ab^	0.49 ^bc^	3.54 ^d^	11.61 ^d^	13.63 ^d^
T5	4.36 ^b^	2.18 ^ab^	2.47 ^b^	0.42 ^ab^	0.61 ^ab^	0.59 ^abc^	3.24 ^e^	9.63 ^ab^	14.22 ^d^
T6	3.58 ^ac^	2.63 ^b^	2.16 ^bc^	0.39 ^ab^	0.57 ^b^	0.48 ^bc^	5.68 ^f^	12.88 ^e^	12.72 ^c^
T7	4.23 ^bc^	2.41 ^b^	2.62 ^b^	0.36 ^b^	0.58 ^b^	0.45 ^c^	5.81 ^g^	10.79 ^c^	13.75 ^d^
SEM	0.20	0.09	0.15	0.02	0.04	0.04	0.38	0.51	0.65
F-value	6.20	2.09	6.71	1.51	1.46	3.89	38.84	26.98	51.13
LSD (*p* = 0.05)	0.66	0.48	0.47	0.16	0.27	0.17	0.04	0.79	0.73

MDA, malondialdehyde; ^nc^ = negative control; T1 = without Carrabiitol^®^; T2, T3, and T4 = seed treatment and foliar application at 2–3 leaf stage with 1 mL/L, 2 mL/L, and 3 mL/L Carrabiitol^®^, respectively, + stress at flowering stage; T5, T6, and T7 = seed treatment and foliar application at pre-flowering stage with 1 mL/L, 2 mL/L, and 3 mL/L Carrabiitol^®^, respectively, + stress at flowering stage. Means followed by a common letter are not significantly different (LSD test, *p* < 0.05). NS—not significant.

**Table 9:** Eigenvalues, variability (%), and cumulative (%) of the PC1 and PC2 axes of PCA.

	Flooding		Salinity		High Temperature		Drought	
	PC1	PC2	PC1	PC2	PC1	PC2	PC1	PC2
Eigenvalues	7.53	1.84	6.78	2.58	7.15	2.87	7.39	2.20
Variability (%)	62.76	15.35	56.54	21.56	59.61	23.99	61.59	18.38
Cumulative (%)	78.10		78.11		83.60		79.97	

## Data Availability

The original contributions presented in the study are included in the article, further inquiries can be directed to the corresponding author/s.
